# C-Reactive Protein Concentration Predicts Change in Body Mass Index during Childhood

**DOI:** 10.1371/journal.pone.0090357

**Published:** 2014-03-06

**Authors:** Barbara H. Lourenço, Marly A. Cardoso, for the ACTION Study Team

**Affiliations:** 1 Public Health Nutrition Program, School of Public Health, University of São Paulo, São Paulo, Brazil; 2 Department of Nutrition, School of Public Health, University of São Paulo, São Paulo, Brazil; University of Sevilla, Spain

## Abstract

**Objective:**

Inflammation may constitute an underlying mechanism for increased risk of developing chronic diseases in later years, but few prospective studies have assessed the influence of low-grade inflammation on body weight gain, particularly among children in low- to middle-income settings with lower prevalence of overweight and obesity. We aimed to investigate whether C-reactive protein (CRP), as a biomarker of low-grade inflammation, predicts changes in body mass index-for-age *z* scores (BAZ) during childhood.

**Methods:**

A population-based longitudinal study was conducted in the Brazilian Amazon among children aged ≤10 years in 2007, with follow-up visits in 2009 and 2012. Outcome was annual change in BAZ. As the main exposure of interest, CRP concentrations were divided into four categories, with values <1 mg/L divided in tertiles plus a fourth category with values ranging from 1 to 10 mg/L. Children were simultaneously screened for iron and vitamin A deficiencies, diarrhea, and wheezing. We used mixed-effect linear regression models to measure the effect of CRP concentrations on annual BAZ change and linear regression models to explore CRP predictors at baseline.

**Results:**

At baseline, 1007 children had CRP and anthropometric data [mean (SD) age: 5.3 (2.9) years; 50.9% male, 84.5% mulatto/mixed-race, 14.0% at risk for overweight or obesity, 4.8% stunted]; 737 were successfully followed up. Morbidities and nutritional deficiencies were widespread. Among participants aged >5 years, children in the highest tertile of CRP <1 mg/L at baseline, regarded as an indicator of low-grade inflammation, had a 0.04 *z*/y higher gain in BAZ (95% CI: 0.01, 0.09 *z*/y) during follow-up. CRP was positively associated with household poverty and worse nutritional indicators.

**Conclusions:**

We found evidence of a role for low-grade inflammation in predicting annual BAZ gain among children aged >5 years.

## Introduction

A state of low-grade inflammation, as measured by biomarkers as C-reactive protein (CRP), can be promoted by exposure to adverse events such as pathogenic agents, psychosocial stress, inadequate sleep, and poor diet [1]–[4], and has also been associated with increased adiposity and cardiometabolic risk [4], [5]. The persistent activation of inflammatory pathways has been proposed as a possible underlying mechanism for increased risk of developing chronic diseases, for which weight gain could be a marker of cumulative environmental derangements, such as cycles of infection and malnutrition, poor living conditions, social disparities, among others [6], [7].

Few longitudinal studies to date, however, have assessed inflammatory status as a possible predictor of future changes in body weight. The ARIC Cohort Study in the United States in men and women aged 45–64 years and the Malmö Preventive Study Cohort in Sweden in men aged 38–50 years observed larger weight gains over three- and six-year periods, respectively, among individuals with higher concentrations of inflammatory markers at baseline [8], [9]. A population-based study conducted in Germany in adults aged 25–74 years found the adjusted odds ratio for a mean annual weight gain of approximately 1 kg/year over a 10-year period was 1.45 for those in the highest quartile of CRP concentrations at baseline, with similar findings for fibrinogen and white blood cell counts [10]. In children, a nationally representative cross-sectional analysis in the United States demonstrated that, although more than 75% of those aged 1–17 years had CRP values below 1 mg/L, there were significant linear trends for the prevalence of higher CRP concentrations with increasing age and body mass, from age 3 years [11]. Prospective analyses using birth cohorts in Finland, Brazil, and India lacked data on markers of inflammation at young ages but identified associations between weight gain over the life course and CRP concentrations in adulthood [12]–[14].

Low- to middle-income regions worldwide experience wide social and health inequities, with high rates of childhood morbidities and deficiencies for nutrients such as iron and vitamin A from early life [15]–[17], resulting in potential consequences for physical development, immunity, and inflammatory status. Concomitantly, these regions are undergoing significant economic and dietary changes, leading to the emergence of excessive weight gain as a public health concern, particularly among the young [18]–[19]. Thus, the identification of early determinants of weight gain is an important step towards understanding the role of low-grade inflammation on body weight. Our objective was to prospectively investigate the association between CRP concentrations at recruitment and change in body mass index (BMI) during childhood in a population-based cohort study in the Brazilian Amazon. We hypothesized that a low-grade inflammation status beginning in childhood could impact increasing weight within a transitional scenario where the lack of resources is coupled with rising overnutrition.

## Methods

### Study population and field procedures

In December 2007, a baseline population-based cross-sectional study on child health and nutrition was conducted in the urban area of Acrelândia (11520 inhabitants, 44% in the urban area), in the Brazilian Amazon region, as previously described [17], [18]. Briefly, alongside local teams from the Family Health Program of the Brazilian Ministry of Health, all 749 households with children aged ≤10 years were identified and invited to participate in the study, of which 13 declined participation and two could not be reached. We initially enrolled 1225 children living in 734 households (98.0% of those identified). Follow-up assessments were carried out in December 2009 for all participants included at baseline, and in July 2012 for children aged >6 years at the time of this last visit. We identified 909 children (74.2%) in 2009 and 514 children out of 714 eligible participants (72.0%) in 2012.

At baseline [17], household interviews with each participant's mother or guardian collected information on child's sex, age and race/ethnicity, birth weight, and occurrence of diarrhea or wheezing in the previous 15 days. The presence of 12 household assets was assessed to generate a wealth index through principal component analysis, and maternal characteristics (educational level and age) were also recorded. For children aged >4 years, data from a validated food frequency questionnaire were used to produce a food frequency index for fruit and vegetable consumption ranging from 0 (lowest category, including children who did not consume vegetables, but consumed fruit ≤3 times/week) to 4 (highest category, including children who consumed vegetables and fruits ≥1 time/day) [20], [21]. A sample (5 mL) of fasting venous blood was collected from children; serum and plasma samples were shipped to São Paulo on dry ice and frozen at −70°C until further analysis.

At all study assessments, children's anthropometric measurements were obtained by trained research assistants using standardized procedures and calibrated equipment [22]. BMI was computed as weight (kg)/length or height (m^2^). BMI-for-age (BAZ) and height-for-age (HAZ) *z* scores were calculated using the World Health Organization (WHO) Child Growth Standards [23] for children ≤5 years and the WHO Growth Reference Data [24] for children >5 years. According to the WHO growth curves, risk of overweight or obesity was defined as BAZ>1 and stunting as HAZ<-2 [23], [24]. Pubertal development according to Tanner stages was ascertained during follow-up examinations conducted in 2009 and 2012 [25].

Written informed consent for participation was obtained before enrollment from parents or guardians of all participating children. The study was approved by the ethical review board of the School of Public Health, University of São Paulo, Brazil.

### Laboratory methods

At baseline, whole blood aliquots in EDTA-containing vacuum tubes were used to perform full blood cell counts and measure hemoglobin on an automated cell counter (Horiba ABX Micro 60, Montpellier, France). Plasma CRP was measured using a high-sensitivity chemiluminescent assay (DPC Immulite, Los Angeles, CA, USA). Plasma ferritin and soluble transferrin receptor were measured with enzyme immunoassays (Ramco, Houston, TX, USA) to determine iron deficiency (soluble transferrin receptor >8.3 mg/L, or ferritin <12 µg/L for children aged up to 5 years or <15 µg/L for those above 5 years) [26). Serum vitamin A (retinol) was measured using standard HPLC methods [27], to determine vitamin A deficiency (<0.70 µmol/L) [28].

### Statistical analyses

Our primary outcome was defined as the change in BAZ during follow-up (*z*/y), while CRP was the main exposure of interest. Complete CRP and anthropometric data were available for 1048 children (85.6%) at baseline. We excluded 41 children with CRP concentrations >10 mg/L from all analyses because these concentrations have been usually associated with active inflammatory processes (as shown in Figure S1 in File S1). Based on the distribution and cut-off points for CRP concentrations used in a large representative survey of children in the United States [11], baseline plasma CRP concentrations below 1 mg/L were categorized as tertiles to assess the influence of low-grade inflammation on BAZ change, while a fourth category contained values ranging from 1 to 10 mg/L. Although there is insufficient evidence to date to define a cut-off related to health outcomes in children, the abovementioned study reported that more than 75% of children and adolescents aged 1–17 years had CRP values below 1 mg/L [11]. In the present study, we referred to the lowest tertile of CRP values <1 mg/L as the reference category, while the highest tertile of CRP<1 mg/L was regarded as an indicator of low-grade inflammation. Other child health indicators at baseline including vitamin A and iron deficiencies, and occurrence of diarrhea or wheezing were also considered as explanatory variables.

The distribution of BAZ was compared by categories of baseline characteristics, using tests for linear trend for ordinal predictors and the Wilcoxon's rank-sum test for dichotomous predictors. Changes in BAZ during follow-up were estimated from mixed-effect linear regression models with an unstructured covariance matrix and random effects for the intercept and slope. For each category of the exposure variables, these models provided estimates for the mean differences in BAZ at the first age of assessment and a coefficient for the interaction term with age representing the mean difference in annual BAZ change. These methods inherently adjust for baseline anthropometric status by clustering repeated outcome measurements within individuals and do not require equal numbers of measurements at exactly the same time points on every participant, allowing for the inclusion of all available measurements. In preliminary analyses, separate models for each child health indicator were adjusted for child's sex and age. Next, we fitted a multiple mixed-effect model with CRP and all other health indicators, while further adjusting for household wealth, maternal age, birth weight, and HAZ at baseline. Other potential covariates were not significantly associated with the outcome and did not affect the estimates of association with children's BAZ. Missing observations (<8%) were included in the multiple models by creating missing-value categories. We also estimated changes in BAZ separately for girls and boys and associations with the exposures of interest remained similar in terms of direction and magnitude; besides, interaction terms with child's sex were not statistically significant. Therefore, we present results for girls and boys altogether. Finally, we explored baseline factors potentially associated with log-transformed CRP concentrations using linear regression models.

We divided children in two age groups (≤5 years and >5 years at baseline) in all analyses. For the older age group only, an additional CRP measurement was available in 2009; therefore, as detailed in File S1, supplemental analyses with a combined CRP score, accounting for the 2007 and 2009 measurements (as a proxy of chronic CRP status) were performed in this sub-sample of our study. We used STATA 11.2 (StataCorp, College Station, TX, USA) for descriptive analyses and SAS 9.3 (SAS Institute, Cary, NC, USA) for mixed-effect linear regression models.

## Results

General child characteristics at each study assessment are presented in Table 1. At baseline in 2007, among 1007 participants with complete anthropometry data and CRP concentrations <10 mg/L, 50.9% were boys and 84.5% mulatto. Distribution of BAZ according to baseline characteristics and age groups are shown in Table 2. In the group of 469 children aged ≤5 years [mean age (SD): 2.7 (1.4) years], the mean BAZ was 0.24 (1.01); 15.4% of girls and 24.1% of boys were at risk for overweight and obesity. Vitamin A and iron deficiencies affected 12.5% and 65.2% of the children, respectively (16.4% anemia due to iron deficiency) while 31.8% reported episodes of diarrhea up to 15 days prior the baseline interview. BAZ was significantly higher in boys than in girls, in children with higher birth weight, and among those with iron deficiency. On the other hand, BAZ was significantly lower among children with diarrhea when compared to those without diarrhea. Among the 538 children aged >5 years at baseline [mean age: 7.6 (1.5) years], the mean BAZ was equal to −0.30 (0.99), with 8.7% of girls and 9.2% of boys at risk for overweight and obesity. Vitamin A and iron deficiencies affected 14.6% and 26.6% of the children, respectively (2.8% with iron deficiency anemia). BAZ was significantly higher in males than in females, in children with higher birth weight, higher HAZ, and higher CRP concentrations (Table 1).

**Table 1:** General characteristics of urban Amazonian children at each study assessment.

		2007 (*n* = 1007)^a^	2009 (*n* = 737)^a^	2012 (*n* = 401)^a^
Sex, *n* (%)	Female	494 (49.1)	374 (50.7)	207 (51.6)
	Male	513 (50.9)	363 (49.3)	194 (48.4)
Age (y), *mean (SD)*		5.3 (2.9)	7.3 (2.9)	10.6 (2.3)
Race/ethnicity, *n* (%)	White	94 (10.1)	71 (10.4)	41 (10.9)
	Mulatto	790 (84.5)	582 (85.5)	315 (84.3)
	Black	51 (5.4)	28 (4.1)	18 (4.8)
BMI-for-age *z* score^b^, *mean (SD)*		−0.05 (1.03)	0.08 (1.13)	0.07 (1.25)
Risk for overweight, *n* (%)	No	866 (86.0)	621 (84.3)	336 (83.8)
	Yes	141 (14.0)	116 (15.7)	65 (16.2)
Height-for-age *z* score^b^, *n* (%)	<−2.0	48 (4.8)	33 (4.5)	12 (3.0)
	−2.0 to −1.1	194 (19.3)	159 (21.6)	75 (18.7)
	−1.0 to 0.9	667 (66.2)	483 (65.5)	272 (67.8)
	≥1.0	98 (9.7)	62 (8.4)	42 (10.5)
Tanner stage, *n* (%)	Pre-pubertal	-	553 (80.0)	199 (49.8)
	Pubertal	-	138 (20.0)	201 (49.2)

a Totals may be less than numbers indicated in brackets for each study assessment because of missing values.

b BMI-for-age and height-for-age *z* scores calculated according to the WHO Child Growth Standards for children ≤5 years and the WHO Growth Reference Data for children >5 years.

**Table 2:** Mean BMI-for-age *z* score in urban Amazonian children with complete C-reactive protein and anthropometric information, according to age groups and baseline characteristics (2007).

		Children ≤5 years at baseline			Children >5 years at baseline		
		*n* (%)^a^	Mean BAZ (SD)^b^^,^ c	*P* d	*n* (%)^a^	Mean BAZ (SD)^b.c^	*P* d
Child's sex	Female	228 (48.6)	0.12 (0.89)	0.01	266 (49.4)	−0.40 (0.95)	<0.01
	Male	241 (51.4)	0.36 (1.09)		272 (50.6)	−0.20 (1.01)	
Child's race/ethnicity	White	47 (10.6)	0.26 (0.90)	0.74	47 (9.6)	−0.37 (1.11)	0.76
	Mulatto	374 (84.0)	0.25 (1.04)		416 (84.9)	−0.28 (0.99)	
	Black	24 (5.4)	0.15 (0.94)		27 (5.5)	−0.32 (1.02)	
Household wealth index	Below median	236 (50.3)	0.18 (0.96)	0.36	259 (48.2)	−0.37 (0.91)	0.10
	Above median	233 (49.7)	0.30 (1.05)		278 (51.8)	−0.24 (1.05)	
Maternal education	≤4 years	152 (33.7)	0.18 (0.94)	0.16	223 (42.8)	−0.36 (0.90)	0.09
	5–8 years	140 (31.0)	0.21 (1.00)		154 (29.6)	−0.30 (1.04)	
	≥9 years	159 (35.3)	0.34 (1.06)		144 (27.6)	−0.18 (1.09)	
Maternal age	≤21 years	75 (16.0)	0.24 (1.11)	0.97	16 (3.0)	0.21 (1.10)	0.56
	22–34 years	318 (67.8)	0.24 (0.92)		362 (67.3)	−0.32 (0.99)	
	≥35 years	76 (16.2)	0.24 (1.23)		160 (29.7)	−0.30 (0.96)	
Birth weight	≤2500 g	24 (5.5)	0.03 (0.81)	<0.01	31 (6.5)	−0.37 (1.31)	<0.01
	2501–3500 g	263 (60.0)	0.10 (1.01)		284 (59.0)	−0.41 (0.94)	
	>3500 g	151 (34.5)	0.52 (0.98)		166 (34.5)	−0.03 (1.01)	
Height-for-age *z* score^c^	<−2.0	30 (6.4)	0.20 (0.97)	0.06	18 (3.4)	−0.37 (0.95)	<0.01
	−2.0 to −1.1	104 (22.2)	0.06 (0.81)		90 (16.7)	−0.50 (0.91)	
	−1.0 to 0.9	287 (61.2)	0.28 (1.02)		380 (70.6)	−0.30 (0.97)	
	≥1.0	48 (10.2)	0.41 (1.25)		50 (9.3)	0.13 (1.14)	
Food frequency index for fruit and vegetable consumption^e^	Lowest consumption (score 0)	-	-	-	64 (12.3)	−0.28 (1.00)	0.66
	Intermediate consumption (score 1–2)	-	-		281 (53.7)	−0.27 (1.00)	
	Higher consumption (score 3–4)	-	-		178 (34.0)	−0.33 (0.97)	
C-reactive protein^f^	1^st^ tertile	129 (27.5)	0.21 (1.03)	0.39	149 (27.7)	−0.47 (0.89)	<0.01
	2^nd^ tertile	96 (20.5)	0.20 (1.01)		124 (23.0)	−0.34 (0.94)	
	3^rd^ tertile	107 (22.8)	0.20 (0.97)		138 (25.7)	−0.24 (0.94)	
	>1 mg/L	137 (29.2)	0.32 (1.02)		127 (23.6)	−0.10 (1.15)	
Vitamin A deficiency	No	386 (87.5)	0.27 (1.03)	0.85	450 (85.4)	−0.32 (0.98)	0.14
	Yes	55 (12.5)	0.26 (0.86)		77 (14.6)	−0.10 (1.02)	
Iron deficiency	No	163 (34.8)	0.03 (0.88)	<0.01	395 (73.4)	−0.31 (0.98)	0.81
	Yes	306 (65.2)	0.35 (1.05)		143 (26.6)	−0.25 (1.03)	
Diarrhea, past 15 days	No	317 (68.2)	0.29 (0.99)	0.05	452 (85.0)	−0.31 (1.01)	0.65
	Yes	148 (31.8)	0.12 (1.03)		80 (15.0)	−0.26 (0.91)	
Wheezing, past 15 days	No	389 (84.4)	0.24 (1.02)	0.72	499 (94.0)	−0.31 (0.99)	0.36
	Yes	72 (15.6)	0.24 (0.97)		32 (6.0)	−0.14 (0.97)	

a Totals may be less than 469 for children ≤5 years and less than 538 for children >5 years at baseline because of missing values.

b Results are mean BMI-for age *z* score (BAZ) and standard deviation (SD).

c BMI-for-age and height-for-age *z* scores calculated according to the WHO Child Growth Standards for children ≤5 years and the WHO Growth Reference Data for children >5 years.

d Test for linear trend for ordinal predictors; for dichotomous predictors, Wilcoxon rank-sum test.

e Information on food frequency index for fruit and vegetable consumption was available for children >4 years only.

f C-reactive protein was categorized as tertiles below 1 mg/L and >1 mg/L. Categories were distributed as follows: 1^st^ tertile: 0.01–0.15 mg/L; 2^nd^ tertile: 0.16–0.38 mg/L; 3^rd^ tertile: 0.39–1.00 mg/L; >1 mg/L: 1.01–9.81 mg/L. Children with C-reactive protein values >10 mg/L were excluded from the analyses (*n* = 41).

A total of 737 children with baseline data were evaluated in 2009, of which 401 were assessed again in 2012. The median follow-up period was 4.6 years (range: 1.7–4.7 years), during which time each child contributed a median of three anthropometric measurements (336 children had two and 401 children had three anthropometric measurements). Children lost to follow-up were not different from those included in analyses, except for household wealth and serum vitamin A concentrations. Among those children successfully followed-up, 21.6% were in the lowest quartile of household wealth compared with 29.6% among those lost to follow-up. Children with follow-up data exhibited a mean vitamin A concentration of 1.25 (0.50) µmol/L, which was higher than the mean 1.16 (0.54) µmol/L among children lost to follow-up.

Mean BAZ at baseline was significantly higher among children aged ≤5 years with iron deficiency (adjusted difference: 0.55 *z*; 95% CI: 0.26–0.85 *z*), but significant associations between baseline health indicators and annual change in BAZ in this age group were not detected (Table 3). Among children aged >5 years, there was no significant difference in the mean BAZ at baseline according to categories of exposure variables. Nevertheless, children in this age group falling within the second and third tertiles of CRP concentrations below 1 mg/L had a larger annual gain in BAZ of 0.04 *z*/y (95% CI: 0.00–0.09 *z*/y in second tertile, and 95% CI: 0.01–0.09 *z*/y in third tertile) when compared with children in the first tertile (Table 3).

**Table 3:** Differences in BMI-for-age *z* score change per year over childhood among urban Amazonian children (2007–2012), according to age groups and baseline health indicators.

		Children ≤5 years at baseline			Children >5 years at baseline		
		n (%)^a^	Unadjusted difference in BAZ change per year (95% CI)^b^^,^ c	Adjusted difference in BAZ change per year (95% CI)^b^^,^ c	n (%)^a^	Unadjusted difference in BAZ change per year (95% CI)^b^^,^ c	Adjusted difference in BAZ change per year (95% CI)^b^^,^ c
C-reactive protein^d^	1^st^ tertile	93 (26.9)	Reference	Reference	113 (28.9)	Reference	Reference
	2^nd^ tertile	76 (22.0)	−0.03 (−0.11, 0.05)	−0.01 (−0.09, 0.07)	84 (21.5)	**0.04 (0.00, 0.09)**	**0.04 (0.00, 0.09)**
	3^rd^ tertile	79 (22.8)	−0.04 (−0.12, 0.04)	−0.04 (−0.12, 0.04)	104 (26.6)	**0.04 (0.00, 0.09)**	**0.04 (0.01, 0.09)**
	>1 mg/L	98 (28.3)	**−0.08 (−0.15, −0.01)**	−0.07 (−0.15, 0.00)	90 (23.0)	0.02 (−0.03, 0.07)	0.02 (−0.03, 0.08)
Vitamin A deficiency	No	286 (88.3)	Reference	Reference	338 (88.7)	Reference	Reference
	Yes	38 (11.7)	0.04 (−0.05, 0.13)	0.06 (−0.03, 0.15)	43 (11.3)	−0.03 (−0.10, 0.04)	−0.02 (−0.09, 0.05)
Iron deficiency	No	123 (35.5)	Reference	Reference	279 (71.4)	Reference	Reference
	Yes	223 (64.5)	−0.05 (−0.10, 0.00)	−0.05 (−0.11, 0.00)	112 (28.6)	−0.02 (−0.06, 0.02)	−0.02 (−0.06, 0.03)
Diarrhea, past 15 days	No	242 (70.6)	Reference	Reference	318 (82.6)	Reference	Reference
	Yes	101 (29.4)	0.01 (−0.05, 0.06)	0.01 (−0.04, 0.07)	67 (17.4)	0.04 (−0.01, 0.08)	0.04 (−0.01, 0.08)
Wheezing, past 15 days	No	289 (85.0)	Reference	Reference	360 (93.8)	Reference	Reference
	Yes	51 (15.0)	0.02 (−0.06, 0.10)	0.04 (−0.04, 0.11)	24 (6.2)	0.02 (−0.09, 0.13)	0.02 (−0.09, 0.13)

a Totals may be less than 346 for children ≤5 years and less than 391 for children >5 years at baseline because of missing values. Missing observations among the covariates were included in the multiple models by creating missing-value categories.

b BMI-for-age *z* scores (BAZ) were calculated according to the WHO Child Growth Standards for children ≤5 years and the WHO Growth Reference Data for children >5 years.

c Mean differences in BAZ change per year and their 95% confidence intervals (CI) were from mixed-effect linear regression models. For each age group, unadjusted differences refer to preliminary models that included each child health indicator with adjustment for sex. Fully adjusted differences were estimated from models including CRP and all other health indicators with further adjustment for household wealth, maternal age, birth weight, and HAZ at baseline. *P*<0.05 for results in bold.

d C-reactive protein categories were distributed as follows: 1^st^ tertile: 0.01–0.15 mg/L; 2^nd^ tertile: 0.16–0.38 mg/L; 3^rd^ tertile: 0.39–1.00 mg/L; >1 mg/L: 1.01–9.81 mg/L.

As shown in the Table S1 in File S1, the association between CRP and weight gain was further explored for participants aged >5 years with CRP measurements available in both 2007 and 2009. Children in the second and third tertiles of CRP concentrations below 1 mg/L in both assessments experienced a 0.05 *z*/y higher increase in BAZ at follow-up compared with those with the lowest CRP concentrations (95% CI: 0.01–0.09 *z*/y). Children with CRP>1 mg/L in 2007 and/or 2009 also had a 0.05 *z*/y higher increase in BAZ (95% CI: 0.01–0.10 *z*/y).

To further elucidate the associations with CRP concentrations, we investigated possible baseline predictors of log-transformed CRP (Table 4). For both age groups, models accounting for approximately 8% of the variance showed a significant association between log-CRP and worse nutritional and socioeconomic indicators. Higher CRP at baseline was associated with lower household wealth, lower serum vitamin A concentrations, and higher values of soluble transferrin receptor. Among children aged >5 years, higher CRP was also significantly associated with higher ferritin concentrations, which can be regarded as a marker of cellular responses to infection and injury.

**Table 4:** Multiple linear regression analysis of baseline predictors of log-transformed C-reactive protein (mg/L) among urban Amazonian children.

Independent variables	Children ≤5 years at baseline^a^	Children >5 years at baseline^a^
	β (95% CI)^b^	β (95% CI)^b^
Male sex	−0.35 (−0.76, 0.06)	-
Household wealth (continuous)	**−0.07 (−0.12, −0.01)**	**−0.06 (−0.11, −0.01)**
Serum vitamin A (µmol/L)	**−0.60 (−0.98, −0.22)**	**−0.70 (−1.07, −0.34)**
Log soluble transferrin receptor (mg/L)	**0.98 (0.36, 1.60)**	**1.15 (0.40, 1.90)**
Log ferritin (µg/L)	-	**0.35 (0.09, 0.60)**
Diarrhea in the past 15 days (yes)	0.42 (−0.03, 0.87)	-
Food frequency index for fruit and vegetable consumption (continuous)	-	−0.12 (−0.26, 0.02)

a Totals are 438 for children ≤5 years and 511 for children >5 years at baseline because of missing values.

b β coefficients and their 95% confidence intervals (CI) were from linear regression models. *P*<0.05 for results in bold.

## Discussion

This longitudinal population-based study was conducted in the Brazilian Amazon region, where childhood morbidities and nutritional deficiencies were widespread. Within this context we observed a positive association between low-grade inflammation detected using CRP tertiles <1 mg/L and annual BAZ gain among older children.

It is widely recognized that children's susceptibility to disease during the first years of life can have short-term consequences for growth and nutrition [29]. We could not detect associations between baseline CRP concentrations and change in BAZ in children aged ≤5 years, but in our study the prevalence of nutritional deficiencies and diarrhea were especially elevated among younger children and were associated with CRP concentrations. Alongside the negative association observed between CRP concentrations and household wealth, these findings could be interpreted as a possible indication of the health burden associated with adverse living conditions. In a recent study, lower maternal education as a measure of socioeconomic position was associated with higher CRP concentrations during childhood, with similar results for family income, paternal education, and the head of the household's occupational class [30].

Research in high morbidity contexts conceives that the continued exposure to infectious diseases, even in the absence of clinical symptoms, may have a cumulative effect on later development and metabolic function [29], [31]. Under chronic activation of the immune system, immediate responses to infections such as increases in CRP concentrations can become maladaptive in the long term. These enduring influences have been proposed as a “cohort morbidity phenotype”, which supports the notion that inflammatory processes persist from early age into adult life [6]. Of note, outcomes observed for the younger group in our analysis do not necessarily mirror the older group's social and health background, but previous reports on child health and nutrition in the same region do not indicate that better structural conditions had existed a decade ago [32], and the prevalence of nutritional deficiencies and morbidities among children in the older age group was also relatively high. We may speculate, therefore, that children aged >5 years residing in the Brazilian Amazon have been exposed to these adverse living conditions for a longer period, with downstream consequences for their CRP concentrations and future BAZ gain.

In a cross-sectional analysis from the United States, where the prevalence of overweight and obesity in children and adolescents is higher [33], a relationship between adiposity and increasing CRP was observed from 3 years of age [11]. In our study, there was no difference in mean adjusted BAZ at baseline according to categories of exposure variables given the lower rate of excessive weight; though this figure has risen in the past decade [34]. In children aged >5 years, we were able to identify significantly higher increases in BAZ during follow-up among those in the higher tertiles of CRP concentrations below 1 mg/L in 2007, indicating a state of low-grade inflammation. In addition, in supplemental analysis we verified larger BAZ gain among those who maintained these higher CRP concentrations between the 2007 and 2009 assessments. Analogously, morbidity data collected in the 1970s for the INCAP Longitudinal Study in Guatemala showed that the number of years that participants experienced serious illness during childhood was positively related to the risk for high waist circumference and obesity 30 years later [31], although no specific measurements of inflammatory markers were available.

Our data do not dispute previous investigations on the role of excessive adiposity in elevating concentrations of inflammatory markers such as CRP via secretion of cytokines. Instead, the present results provide further evidence of the potential factors related to weight gain during childhood. Both positive and negative associations found between low-grade inflammation and change in body weight seem to be implicated in disrupted metabolic outcomes. Further studies, however, are still needed to elucidate these pathways.

Among the possible mechanisms for increased weight gain due to low-grade inflammation, several hypotheses integrate the hypothalamic-pituitary-adrenal axis and point to a close connection between nutrition and immunity. Imbalanced energy intake and storage can be a result of the expression of anti-inflammatory mediators such as glucocorticoids in response to the low-grade inflammatory stimulus, possibly leading to weight gain through the upregulation of neuropeptide Y secretion [35]. Leptin, whose secretion is upregulated by pro-inflammatory cytokines as interleukin-6 and tumor necrosis factor-α during acute inflammation, is suggested as a key link between neuroendocrine functions, the immune system, and nutritional status. However, chronic inflammation results in lower leptin concentrations, probably affecting appetite and food intake control while increasing susceptibility to infectious diseases [36], [37]. In particular, CRP is considered a major leptin-interacting protein that may impair leptin signaling and promote leptin resistance [38]. While previous studies have found an association between markers of low-grade inflammation (including CRP and leptin) and dietary intake of fats and antioxidant vitamins in children aged 6-14 years [39], our study found that CRP concentrations in children aged >5 years were negatively associated with vitamin A concentrations and frequency scores for fruit and vegetable consumption. With the progressive replacement of minimally processed and traditional food items by industrialized food products in settings undergoing a nutrition transition [40], mechanisms for weight gain can be increasingly exacerbated by a “pro-inflammatory” diet characterized by high energy density, low fiber, in addition to the consumption of more saturated fat, sodium and added sugar.

Our study has some limitations. Although the overall follow-up rate was high, children lost to follow-up were predominantly from poorer households and presented lower vitamin A concentrations. Although attrition is common in prospective studies, its implications are difficult to assess. Associations might have been underestimated because children with worse health and socioeconomic indicators were lost to follow-up. The statistical methodology enabled us to include all existing outcome measurements from the participants to minimize the impact of attrition, but caution should be taken when extrapolating our findings to the general population. Also, we lacked data on use of nutritional supplements and were unable to further explore their relationship with CRP or weight gain. Major strengths of our study include its longitudinal design and use of direct anthropometric measurements. In contrast to the majority of prospective investigations in children, inflammatory status was assessed at baseline, with repeated CRP measurements in a sub-sample to account for chronic CRP status over five years. In addition, we used a broad range of covariates including nutritional deficiencies, morbidities, pubertal development, and nutritional indicators to control for possible confounding.

It is noteworthy that, although magnitude of change in BAZ per year among children in the second and third tertiles of CRP<1 mg/L was small, our outcome was measured in sex and age-specific WHO *z* scores. Therefore, these estimates of change stand for increases in relation to expected growth patterns. In our study population, we found evidence for a role of low-grade inflammation in predicting annual BAZ gain among children aged >5 years who were followed up for nearly five years, living in a resource-poor setting in which nutritional deficiencies and childhood morbidities are common. Our findings may provide a better understanding of the predictors of weight change in low- to middle-income countries, and such increase in BAZ may impose a concern particularly for eutrophic children in this age group in regions experiencing the epidemiological transition from nutritional deficiencies to excessive weight gain. Considering low-grade inflammation as a possible underlying path, there are important implications for the formulation of public health policies, which should address social and health inequities, with potential benefits at reducing both susceptibility to infection and the chronic disease burden.

## Supporting Information

File S1 **Information on study design and subsample analysis with a combined C-reactive protein score.**
(PDF)
