# Supplementary material for: C-Reactive Protein Concentration Predicts Change in Body Mass Index during Childhood
Source: PLoS One. 2014 Mar 6;9(3):e90357. doi: 10.1371/journal.pone.0090357 (PMC3946086; doi:10.1371/journal.pone.0090357)
Supplement: File S1 — Information on study design and subsample analysis with a combined C-reactive protein score. (PDF) [file pone.0090357.s001.pdf]

Supporting information

Figure S1. Study design and participants at each assessment.

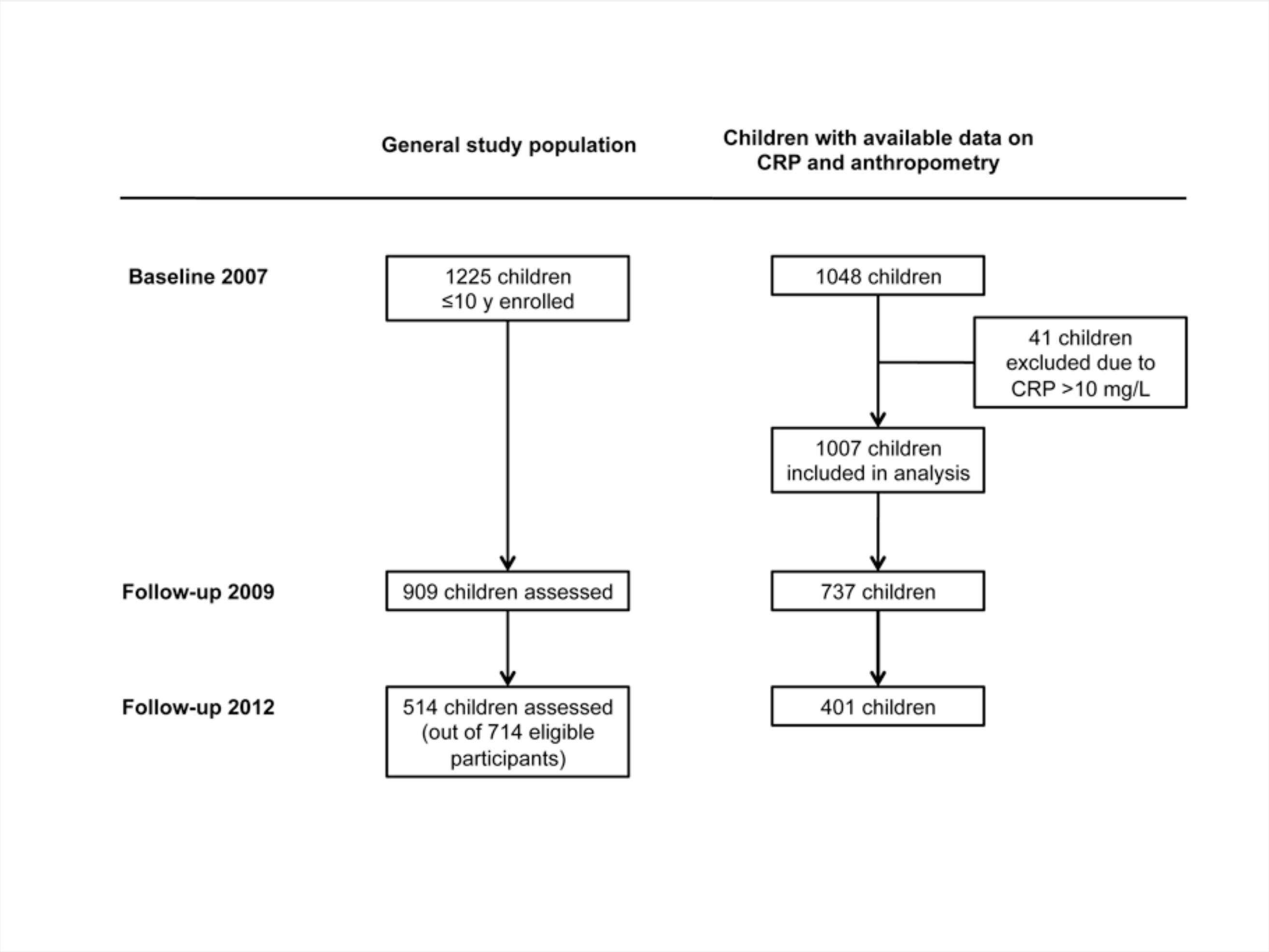

Subsample analysis with a combined C-reactive protein score

The association between CRP and weight gain was further explored for a sub-sample of participants aged >5 years at baseline of the present study, with CRP measurements available in 2007 and 2009.

In the follow-up assessment carried out in December 2009, sociodemographic and anthropometric data were updated for all eligible children enrolled at baseline, and a clinical examination was performed among older participants. After the clinical examination, blood samples from these children were stored away from light and centrifuged within 1 h of collection at the field

laboratory in Acrelândia; serum and plasma aliquots were shipped to São Paulo on dry ice and frozen at -70°C until analysis. Plasma CRP in the 2009 assessment was measured using the same protocol as described for baseline, with a high-sensitivity chemiluminescent assay (DPC Immulite, Los Angeles, CA, USA).

Overall, CRP measurements <10 mg/L in both 2007 and 2009 assessments were available for *n*=349 children aged >5 years at baseline. For both assessments, we classified these CRP levels into tertiles below 1 mg/L plus a fourth category with values ranging from 1 to 10 mg/L. Next, we generated a combined CRP score, as a proxy of chronic CRP status, by allocating participants to three categories as follows: children who were classified in the first tertile in both assessments (reference category), children who were classified in up to the second and third tertiles of CRP levels below 1 mg/L in 2007 and/or 2009, and children who presented CRP >1 mg/L in 2007 and/or 2009.

In statistical analysis for this sub-sample, the combined CRP score was entered as the main exposure variable in mixed-effect linear regression models to estimate changes in BAZ during follow-up, with preliminary adjustment for child's sex. Subsequently, we fitted a multiple mixed-effect model by including the combined CRP score and all other health indicators explored in the present study (vitamin A and iron deficiencies, and occurrence of diarrhea and wheezing, measured at baseline), with further adjustment for household wealth, maternal age, birth weight, and HAZ at baseline. Other potential covariates were not significantly associated or did not affect the estimates of association with children's BAZ. Missing observations (<8%) were included in the multiple model by creating missing-value categories. The results are presented in the Table S1 below.

Table S1. Differences in BMI-for-age *z* score change per year over childhood among urban Amazonian children aged >5 years at baseline (2007-2012), according to combined C-reactive protein score based on measurements from 2007 and 2009.

		n (%)	Unadjusted difference in BAZ change per year (95% CI)[a,b]	Adjusted difference in BAZ change per year (95% CI)[a,b]
Combined C-reactive protein score	1st tertile in both 2007 and 2009	58 (16.6)	Reference	Reference
	2nd to 3rd tertile in 2007 and/or 2009	144 (41.3)	0.05 (0.01, 0.09)	0.05 (0.01, 0.09)
	>1 mg/L in 2007 and/or 2009	147 (42.1)	0.05 (0.01, 0.10)	0.05 (0.01, 0.10)

[a] BMI-for-age *z* scores (BAZ) were calculated according to the WHO Growth Reference Data.
[b] Mean differences in BAZ change per year and their 95% confidence intervals (CI) were from mixed-effect linear regression models. For each age group, unadjusted differences refer to a preliminary model that included the combined CRP score with adjustment for sex. Fully adjusted differences were estimated from models including the combined CRP score and all other health indicators (vitamin A and iron deficiencies, and occurrence of diarrhea and wheezing, measured at baseline), with further adjustment for household wealth, maternal age, birth weight, and HAZ at baseline.
